# The (Bio)Chemistry of Non-Transferrin-Bound Iron

**DOI:** 10.3390/molecules27061784

**Published:** 2022-03-09

**Authors:** André M. N. Silva, Maria Rangel

**Affiliations:** 1 LAQV-REQUIMTE, Departamento de Química e Bioquímica, Faculdade de Ciências, Universidade do Porto, Rua do Campo Alegre s/n, 4169-007 Porto, Portugal; 2 LAQV-REQUIMTE, Instituto de Ciências Biomédicas Abel Salazar, Universidade to Porto, Rua Jorge Viterbo Ferreira N228, 4050-313 Porto, Portugal; mrangel@icbas.up.pt

**Keywords:** iron overload, non-transferrin-bound iron, NTBI, labile plasma pool, LPI, citrate, albumin

## Abstract

In healthy individuals, virtually all blood plasma iron is bound by transferrin. However, in several diseases and clinical conditions, hazardous non-transferrin-bound iron (NTBI) species occur. NTBI represents a potentially toxic iron form, being a direct cause of oxidative stress in the circulating compartment and tissue iron loading. The accumulation of these species can cause cellular damage in several organs, namely, the liver, spleen, and heart. Despite its pathophysiological relevance, the chemical nature of NTBI remains elusive. This has precluded its use as a clinical biochemical marker and the development of targeted therapies. Herein, we make a critical assessment of the current knowledge of NTBI speciation. The currently accepted hypotheses suggest that NTBI is mostly iron bound to citric acid and iron bound to serum albumin, but the chemistry of this system remains fuzzy. We explore the complex chemistry of iron complexation by citric acid and its implications towards NTBI reactivity. Further, the ability of albumin to bind iron is revised and the role of protein post-translational modifications on iron binding is discussed. The characterization of the NTBI species structure may be the starting point for the development of a standardized analytical assay, the better understanding of these species’ reactivity or the identification of NTBI uptake mechanisms by different cell types, and finally, to the development of new therapies.

## 1. Introduction

Iron is an essential micronutrient for almost all living organisms, participating in a variety of metabolic processes. Perhaps the best-known function of iron is its presence in hemoglobin, where it is involved in the transport of oxygen. However, iron plays a crucial role in cellular processes such as DNA synthesis, epigenetic regulation, or cellular respiration [1].

The biological importance of iron results from its adaptable chemistry and natural abundance in the Earth’s crust [2]. Iron can accept or donate electrons, participating in oxidation–reduction reactions, occurring under various oxidation states (from −2 to +6). The most common oxidation states are Fe^3+^, a hard acid that prefers coordinating oxygen atoms, and Fe^2+^, an intermediate acid that favors bonding with ligands containing nitrogen or sulfur atoms [2]. However, these same chemical properties are responsible for iron toxicity. This harmful effect is related to the role played by iron in the Fenton reaction, which can amplify the deleterious effects of oxidative stress [3].

To avoid its hazardous effects, in the human body, iron homeostasis is tightly controlled. Humans do not actively excrete iron, and therefore, maintenance of iron balance involves the control of its absorption, storage, use and distribution. This is mainly achieved by the action of hepcidin, the hormone which controls cellular iron export and its release into circulation [4].

Hemoglobin-bound iron, present in circulating erythrocytes and developing erythroid precursors, represents about two-thirds of the total iron present in the body [5]. Most of the iron required for erythropoiesis comes from the recycling of senescent red cells promoted by macrophages of the reticuloendothelial system. This recycling mechanism is essential, as only 1 to 2 mg of iron are daily absorbed from the diet. The remaining iron in the body is stored in hepatocytes and macrophages, or it is being used for cellular metabolic processes. 

The link between the places of iron absorption, utilization and storage is made by the blood circulation, where iron transport is carried out by transferrin (Tf). Tf is a glycoprotein, produced in the hepatocytes, with two globular domains, each with a binding site with high affinity for the Fe^3+^ ion [6]. Healthy individuals present typical Tf saturation levels around 30%, ensuring a large blood plasma unsaturated iron-binding capacity [7]. Iron-binding by Tf allows its solubilization at physiological pH values and hinders its participation in deleterious redox reactions. Furthermore, Tf functions as an iron addressing system, ensuring that cellular uptake only occurs in cells expressing the transferrin receptor (TfR) and avoiding undesired tissue iron deposition. However, under several pathological conditions, blood serum NTBI has been observed. NTBI has been proposed as a main toxicity mediator, responsible for increased oxidative stress and tissue iron deposition, leading to several comorbidities such as liver damage, endocrinopathies, cardiac disease, atherosclerosis and neurodegeneration [8,9,10,11,12,13,14]. Therefore, NTBI constitutes an important concept for the clinical management of patients [15,16], but lack of knowledge regarding the chemical nature of NTBI has precluded the development of standardized assays and its use as a biochemical marker in clinical practice [15,17].

## 2. NTBI: Non-Transferrin-Bound Iron

NTBI was first described by Hersko et al. as iron which was available for chelation by diethylenetriamine pentaacetic acid in the presence of fully saturated Tf in the serum of thalassemia patients [18]. Since, its presence has been confirmed in transfusional iron overload [19,20] and hereditary hemochromatosis (HH) [21,22,23]. However, the presence of NTBI is not restricted to iron overload diseases and these toxic iron species have been detected in diabetes mellitus, end-stage renal disease, cancer patients undergoing chemotherapy, or anemia patients under iron supplementation regimens [24,25,26,27].

At present, NTBI is defined as blood serum iron not bound to Tf, heme or ferritin [28]. As recently proposed by Cabantchik and Hershko [15], a further distinction should be made between iron associated with endogenous plasma ligands and iron associated with chelators used in therapeutic practices or ferric oxyhydroxy-carbohydrate structures derived from iron supplements. Along with NTBI, different terms are commonly used to refer to blood serum iron that is not bound to Tf. These indicate the chemical heterogeneity of NTBI species and probably reflect the existence of sub-pools showing different lability and redox reactivity [29]. Labile plasma iron (LPI) has been used to describe NTBI species which are readily available to participate in redox cycling and, similarly, directly chelatable iron (DCI) has been used to describe iron which is available to direct chelation by an exogenous ligand [29,30,31,32]. However, NTBI has mostly prevailed as the terminology used for extracellular iron species, while labile iron pool or labile cell iron are commonly used when referring to intracellular labile iron species [29,33].

The most common cause for the appearance of NTBI species will be high Tf saturation. Nevertheless, NTBI has been confirmed in the sera of HH patients with only partially saturated Tf [21,22,23,34]. Currently, it is commonly accepted that NTBI will appear when Tf saturation levels exceed 70% [12], but the presence of NTBI has been confirmed in diabetes patients with Tf saturation below 50% and even in healthy individuals [25,35]. Therefore, the presence of NTBI cannot be regarded as a simple Tf spillover phenomenon, but rather as an expression of the kinetics balance between iron egress into blood serum, binding by Tf, and removal/utilization from circulation [36].

NTBI is taken up by several tissues, but the main organ responsible for its clearance from circulation is the liver, as recently reviewed by Knutson [37], with hepatocytes being particularly effective in its uptake [38,39,40,41,42]. Despite the high efficiency of NTBI extraction by the liver, persistence of these iron species promotes iron deposition in other organs, such as the pancreas, kidney and heart [43,44,45]. Studies in hypotransferrinemic mice, with ^59^Fe, have shown that NTBI was removed from serum with a half-life of 30 s, as opposed to the 50 min required for Tf-bound iron [43]. This greater efficiency in the uptake of NTBI, when compared to Tf-bound iron, seems a common feature for various tissues. Furthermore, contrary to what is observed with Tf, NTBI internalization appears not to be inhibited by cellular iron loading [28,37].

Several proteins have been identified as NTBI transporters (reviewed by Knutson [37]). These proteins include divalent metal transporter 1 (DMT1), ZRT/IRT-like proteins 8 and 14 (ZIP8 and ZIP14), and calcium channels (LTCC, TTCC and VGCC). The relevance of these transporters seems to be cell-type-specific, with NTBI uptake in the hepatocytes and pancreas β-cells mainly occurring through ZIP14 [46,47], in neurons through ZIP8 and VGCC [48], while uptake in cardiomyocytes occurs through LTCC [49,50]. The referred proteins are divalent metal transporters, and an iron reduction step is required prior to NTBI uptake. This observation is supported by the inhibition of cellular Fe^3+^ species uptake in the presence of specific Fe^2+^ chelators [47,51]. The involvement of several cell surface ferrireductases in NTBI transport has been proposed, such as the cytochrome b561 (Cyt b561) family (including Dcytb), Steap proteins, prion protein (PrP), and α-synuclein [37]. Dcytb is expressed at the cell surface of astrocytes [52], and Steap2 colocalizes with Zip8 in neurons [48]. PrP is a ubiquitously expressed cell surface protein, and knock-out studies have indicated that it may play a role in NTBI uptake in the liver, kidney and pancreas [53,54].

Despite these recent advances in the identification of NTBI transporters, the full depiction of NTBI uptake routes will require the chemical characterization of NTBI speciation and the identification of pathophysiological NTBI species.

## 3. The Chemical Characterization of NTBI

Like most extracellular iron species, NTBI is assumed to be constituted by Fe^3+^. The release of iron into the bloodstream occurs through ferroportin, which requires an associated ferroxidase activity [55,56]. Furthermore, the abundance of ceruplasmin, a ferroxidase, in the serum should ensure that iron remains in the Fe^3+^ oxidation state [57]. Contrarily, in the intracellular environment, the predominant iron oxidation state is Fe^2+^ [58]. 

Given the poor solubility of the ferric ion, Fe^3+^, at the serum pH value, NTBI species must consist of iron associated with blood serum components responsible for its binding and solubilization. Low molecular weight (LMW) carboxylic acid compounds, with oxygen donor atoms, are the most likely candidates for NTBI ligands. In fact, early chemical models of iron speciation in the plasma predicted 99% of iron to be associated to citrate, as ferric citrate hydroxide [59]. The involvement of citrate in NTBI composition was later elegantly confirmed by nuclear magnetic resonance (NMR) experiments with sera from HH patients [60]. The comparison between the NMR spectra of plasma samples collected from healthy individuals and patients diagnosed with HH allowed the identification of differences in signal intensities from citrate and acetate. To validate if these differences could be ascertained to iron binding, plasma samples were also incubated with the iron chelator desferrioxamine or supplemented with exogenous Fe^3+^, indicating that spectral differences were indeed resulting from the presence of iron.

Early experiments applying size exclusion chromatography to the serum of hypotransferrinemic mice have indicated the presence of high molecular weight (HMW) NTBI species [61]. The existence of HMW-NTBI species has since been confirmed in the serum from thalassemia patients, with only approximately 10% of NTBI being able to penetrate 30 kDa exclusion ultrafiltration devices [62]. These HMW iron species could consist of polymeric iron species or iron non-specifically associated with serum proteins. Being the most abundant protein in the serum and recognized as a carrier both for organic compounds and metal ions [63,64], human serum albumin (HSA) has been proposed as the most likely protein candidate [65]. In fact, iron binding by albumin has been observed by several in vitro studies, but definitive proof for iron association with this protein *in vivo* has remained elusive [62,66].

## 4. Ferric Citrate Chemistry

Citrate is a ubiquitous iron chelator, acting as a bacterial or plant siderophore, as the main iron transporter in the plant xylem sap, or an NTBI ligand in the blood serum [67,68,69]. Citrate is a typical α-hydroxycarboxylate siderophore [67], capable to coordinate iron by its three carboxylic acid groups and its hydroxyl moiety. In fact, the hydroxyl group is fundamental for iron coordination and may determine the affinity of these iron chelators [70].

**Figure 1:**
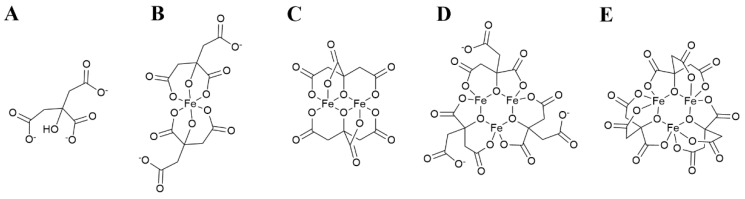
Schematic representation of the structure of citrate and ferric citrate complexes in aqueous solution. (**A**) Citrate anion. (**B**) [FeCit_2_]^5−^. (**C**) [Fe_2_Cit_2_]^2−^. (**D**) [Fe_3_Cit_3_]^3−^. (**E**) alternative structure for [Fe_3_Cit_3_]^3−^. Structures C, D and E are adapted from Gautier-Luneau et al. [71], structure E has been proposed by Fukushima et al. [75].

The structure of citrate (Figure 1A) determines a diversity of coordination modes, most commonly as a tridentate ligand, with coordination of Fe^3+^ occurring through two carboxylate and the hydroxyl moieties [71]. This coordination mode leaves a free carboxylate capable to bind another Fe^3+^ ion, which favors the formation of polymeric complexes. To date, monoferric dicitrate ([Fe(Cit)_2_]^5−^), diferric dicitrate ([Fe_2_(Cit)_2_]^2−^), diferric tricitrate ([Fe_2_(Cit)_3_]^6−^) and nonairon octacitrate ([Fe_9_O(Cit)_8_]^7−^) complexes have been isolated and their crystal structure has been determined [71,72,73,74] (schematic representation in Figure 1B,C). Species with 3 ferric ions ([Fe_3_(Cit)_3_]^3−^ and [Fe_3_O(Cit)_3_]^5−^) have been identified by mass spectrometry [71,75,76] (Figure 1D,E).

Aqueous solution studies, probed by mass spectrometry, indicate that the different ferric citrate complexes co-exist in an intricate chemical equilibrium, with the relative abundance of individual species depending on the pH value and the iron:citrate molar ratio [71,76]. Low ratios (corresponding to high excess citrate) favor formation of [Fe(Cit)_2_]^5−^ while increasing the iron abundance leads to the formation of polynuclear species [Fe_2_(Cit)_2_]^2−^ and [Fe_3_(Cit)_3_]^3−^. This effect combines with medium acidity, with low pH values favoring polynuclear species and a basic environment favoring the mononuclear complex. Under the serum conditions of pH, physiological levels of citrate (80–120 µM) and typical NTBI concentrations (1–10 µM) [Fe(Cit)_2_]^5−^ are the most abundant species with polynuclear [Fe_2_(Cit)_2_]^2−^ and [Fe_3_(Cit)_3_]^3−^ species only existing at relevant concentrations for the higher NTBI values.

The complex speciation of ferric citrate under physiological conditions provides valuable insights for the reactivity and bioavailability of NTBI. Bacteria seem to show a preference for polynuclear ferric citrate complexes, with FecA, the outer membrane ferric citrate transporter from *E. coli*, binding [Fe_2_(Cit)_2_]^2−^ [77,78] and FecC, from *B. cereus*, binding [Fe_3_(Cit)_3_]^3−^ [75]. A similar preference has been described for eukaryotic cells, with isolated T lymphocytes and hepatocytes showing a preference for the uptake of polynuclear species ([Fe_3_(Cit)_3_]^3−^) [39].

A required step for NTBI cellular uptake is Fe^3+^ reduction [39,79,80]. Reduction may be promoted by LMW reducing agents such as ascorbate or by a series of transmembrane ferrireductases [81,82]. The speciation of ferric citrate may be essential in determining NTBI availability to this reduction step. Furthermore, it may impact on the redox activity of NTBI and its ability to promote oxidative damage in the serum and extracellular environment. Although reduction potentials are difficult to determine for the ferric citrate system due to its complex speciation, Vukosava et al. have reported that at pH = 5.5 reduction of [Fe(Cit)_2_]^5−^ occurs at −0.1 V, while reduction of polynuclear complexes is observed at lower reduction potentials (−0.28 V) [83]. Contrastingly, Adam and co-workers reported a reduction potential of *ca.* 0 V (−0.03 V < E°′ > +0.01 V) for Fe^3+^—citrate species at physiological pH, with only small variations resulting from different iron:citrate ratios [84]. Further, the authors have evaluated the ability of ascorbic acid to induce iron redox cycling in the presence of high citrate excess, where the [Fe(Cit)_2_]^5−^ species will predominate. Their results supporting the occurrence of slow redox cycling in the presence of H_2_O_2_, and thus pinpointing Fe^3+^—citrate complexes as potential mediators of oxidative stress.

The speciation of ferric citrate species is also a determinant for the effective chelation of NTBI. Kinetics experiments conducted by Gautier-Luneau et al. have shown that the transfer of iron to external chelators (TRENCAMS or O-TRENSOX) at physiological pH occurs more than 20 times faster for the [Fe(Cit)_2_]^5−^ species than for polynuclear complexes ([Fe_2_(Cit)_2_]^2−^ or [Fe_3_(Cit)_3_]^3−^) [71].

## 5. Iron Binding by Serum Albumin

**Figure 2:**
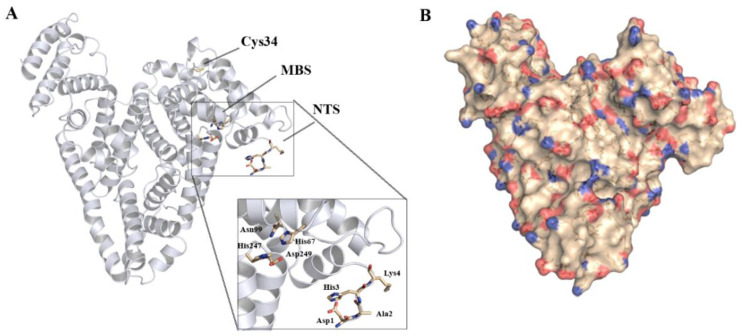
Human serum albumin structure (PDB: 1BM0 [94]). (**A**) Known metal ion binding sites in HSA: NTS (N-terminal site), MBS (Metal Binding Site A) and Cys34 (reduced cysteine at position 34). (**B**) Surface map from HSA, highlighting sidechain oxygen atoms from aspartate and glutamate residues (red) and sidechain nitrogen atoms from arginine and lysine residues (blue). The 4 N-terminal amino acid residues in the protein sequence were absent from the crystallographic structure and were manually modelled for this representation.

HSA is the most abundant protein in the blood plasma, with a concentration around 40 g/L (~600 µM). HSA is a multicarrier protein, with the ability to bind numerous metabolites, fatty acids and also metal ions [63]. The interaction of HSA with divalent metal ions is well described and two binding sites have been characterized (reviewed by Bal et al. [85] and Al-Harthi et al. [63]): the protein N-terminal site (NTS) [86], which has shown a preference for Cu^2+^ and Ni^2+^ binding, and the metal binding site A (MBS-A) [87,88,89,90], at the interface of domains I and II, which shows greater selectivity towards Zn^2+^ and Cd^2+^ (Figure 2A). Additional metal ion binding sites have been proposed, with a putative MBS-B being involved in Zn^2+^ and Cd^2+^ binding [88,89,90]. Coordination at these binding sites involves both nitrogen and oxygen atoms, respectively, from histidine sidechains, the protein backbone or carboxylate residues. Binding of Hg^2+^, Au^+^ and Pt^+^ has been shown to occur at the reduced Cys^34^ residue [91,92,93].

Several reports of iron binding to HSA have also been published, but no specific binding site has been identified. Fe^3+^ present in the blood plasma should favor coordination to oxygen atoms in the carboxylate moieties of aspartate and glutamate sidechains. HSA is rich in ionic amino acids and has a net negative charge of –15 at physiological pH values, with a total of 98 carboxylate moieties (36 Asp and 62 Glu residues), most lying at the protein surface (Figure 2B). It is hypothesized that Fe^3+^ can bind non-specifically to some of these carboxylate clusters, similarly to what has been described for Ca^2+^ [95,96].

Anghileri has shown the possibility to prepare stable iron-HSA complexes from ^59^FeCl_3_ in acetate buffer, probed by paper electrophoresis and gel filtration chromatography [97]. Hershko and co-workers [18] noted that ^59^FeCl_3_ added to thalassemic sera migrated with the HSA fraction in electrophoretic separation. Using, bovine serum albumin (BSA) Loban et al. [98] predicted a total of 9 iron binding sites. However, more recently, employing a fluorescence titration, Xu et al. reported on the existence of only 1 specific binding site, with Kd = 3.46 × 10^−8^ M at 37 °C [99]. The described studies were performed in the absence of an iron ligand, and interference from the likely formation of iron hydroxide species cannot be excluded.

Due to the poor solubility of Fe^3+^ at physiological pH values, studies of iron binding to HSA have also been carried out in citrate media. Coddington and Perkins [100] have demonstrated binding at low pH value, with experiments with acetylated or esterified albumin suggesting the electrostatic association of negatively charged Fe^3+^-citrate complexes to protein positive charged sites. However, with native HSA, binding was suppressed at pH 7 and an iron:citrate molar ratio of 1:5 prevented binding above pH 5. Nevertheless, the authors estimated the existence of 13 iron binding sites. More recently, using gel filtration chromatography, Løvstad [101] has proposed the formation of ternary BSA-Fe^3+^-citrate complexes, with albumin being able to bind 2 to 3 ferric ions. This study was, however, carried out with iron and citrate present in a 1:1 molar ratio, which may promote the formation of iron-hydroxyde-citrate HMW structures [76,102] and may not reflect the presence of physiologically relevant Fe^3+^-citrate species.

More recently, Evans et al. [62] have assessed iron binding by HSA under conditions relevant for NTBI speciation, evaluating physiologically relevant molar ratios of iron:citrate, while maintaining physiologically relevant values for citrate concentration and pH, and iron levels typically found in β-thalassemia major patients. Under such conditions, it has been found that HSA was able to hinder the permeation of ferric citrate complexes through 30 kDa cutoff filtration units, suggesting that it plays a significant role in NTBI speciation. However, in the same study, it proved to be impossible to isolate HSA-bound iron from the sera of β-thalassemia patients and HSA immunoprecipitation did not reduce the detectable NTBI in these serum samples. Subsequently, we have evaluated the interaction of Fe^3+^ and HSA by gel permeation chromatography [66] under physiological concentrations of albumin (40 g/L) and citrate (100 µM). It was shown that with native HSA at low Fe^3+^ concentrations (1 µM), most of the Fe^3+^ available (~60%) associates with the protein fraction, this value decreasing logarithmically to less than 20% at higher iron values (>10 µM). ^14^C-labelled citrate was used to evaluate the formation of ternary complexes, with results suggesting that most of the HSA-bound iron is directly associated to the protein. Furthermore, our results demonstrate that non-enzymatic protein post-translational modifications, such as oxidation or glycation, have the potential significantly increase the ability of HSA to bind iron. These modifications were thought to introduce chelating functional groups to HSA, and even when present at low relative abundance, may have a profound impact in the distribution of NTBI species.

## 6. NTBI Detection and Quantification

Over the years, several methods have been developed to quantify NTBI [29]. The most widely used assay is based on NTBI mobilization by a chelating agent (commonly, nitrilotriacetic acid—NTA) under experimental conditions which limit the mobilization of transferrin-bound iron (TBI) [22,103]. The Fe-NTA complex is thus separated from TBI by molecular weight exclusion ultrafiltration and the iron quantified in the ultrafiltrate.

Alternative methods, such as the bleomycin and the labile iron pool (LPI) assays, take advantage of iron reactivity and depend on NTBI reduction. Bleomycin can act as a selective Fe^2+^ chelator [104]. In the LPI assay, which has been implemented in several laboratories, serum is supplemented with ascorbic acid to initiate the iron dependent Fenton catalyzed oxidation of a fluorescent probe (1,2,3-dihydrorhodamine) [31]. However, both assays tend to give rather low NTBI values, which is generally attributed to them only being able to assess a sub-pool of the heterogeneous NTBI group of species. Furthermore, both seem to being subject to interferences from heme and hemoglobin.

Recently, Ma and co-workers [105] have introduced a fluorescence bead assay which allows direct NTBI detection by flow cytometry. The main advantage of this bead assay would be the simplicity of use, taking advantage of common analytical equipment present in almost any modern hospital. The method has been extensively compared with the NTA-based assay, showing low agreement in their results [106]. Nevertheless, it has shown a lower dependence on Tf saturation, preventing both the existence of false negatives at the lower Tf saturation bound and false positives at high Tf saturation levels.

Two international round robins have been promoted, where samples have been circulated by several laboratories [17,107]. Results show that NTBI values for the same sample vary considerably between assays and even between laboratories reporting on the same method. Used in the most recent study, the bead assay failed to detect NTBI in most samples [17]. Furthermore, both the NTA-based assay and the LPI assay are laborious and seem to show analyst dependence.

The development of a consistent assay is a requirement for NTBI to become an important biochemical biomarker in the clinical management of iron overload and related diseases. It seems that a better understanding of the serum speciation of these elusive iron species is crucial to allow the development of new and more reliable analytical methods.

## 7. NTBI in Iron Related Disorders

NTBI was first identified in iron overload diseases, either HH or hematological iron overload disorders (β-thalassemia major and intermedia) and NTBI uptake is thought to be the major route for iron deposition in several tissues. Liver hepatocytes are particularly efficient in NTBI clearance [40,42,108], which justifies the high liver iron loading observed in HH. The heart and the pancreas are also important sites of iron accumulation in iron overload. Studies with mice have shown that these organs are loaded when the liver approaches its iron loading capacity [109,110]. Iron loading in the pancreas occurs mainly in acinar and β-cells [111,112], the latter being particularly susceptible to iron-promoted dysfunction [113]. Proportionally, cardiomyocytes seem to load less iron than the liver or the pancreas, but they show a preference for NTBI uptake over TBI [114] and their function is significantly hindered by iron loading [115].

NTBI has also been detected in diabetes mellitus (DM) [25,35,116,117] and type 2 DM patients show increased iron stores [118]. The presence of NTBI is thought to be a major contributor to increased oxidative stress both in intracellular and the circulating compartment. Furthermore, NTBI levels seem to correlate with both liver and pancreatic functions, with patients showing increased levels of insulin resistance and decreased insulin production [119,120]. NTBI-generated oxidative stress also seems to play a role in the development of comorbidities in the general population, with recent studies showing a correlation with increased cardiac disease [9] and a mechanistic link with the development of atherosclerosis [10].

These toxic iron species have also been detected in other pathologies such as end-stage renal disease, myelodysplastic syndrome, and during myeloablative therapy and stem cell transplantation [24,28]. Although the pathophysiological role of NTBI in these diseases is less explored, it is believed to promote tissue iron deposition and oxidative stress.

Iron has also been long recognized as an important player in neurodegeneration [14]. Brain iron levels are associated with cognitive decline in Alzheimer’s disease [121] and iron has been shown to colocalize with amyloid β deposits [122,123,124]. Furthermore, iron deposits seem to be involved in the generation of oxidative stress and cell death through ferroptosis [125,126]. Several studies have also related iron accumulation in the substantia nigra of Parkinson patients with disease severity [127,128,129]. Dyshomeostasis of brain iron metabolism is also linked with Friedreich’s ataxia, multiple sclerosis, Huntington’s disease, aceruloplasminemia and neuroferritinopathy [14].

There is no clear evidence that high iron load and the persistent presence of circulating NTBI in HH and β-thalassemia major predispose to neurodegeneration, but recent studies have correlated the prevalence of HFE polymorphisms with brain iron load and homeostasis, suggesting that the brain may not be entirely protected in iron overload conditions [130,131]. Furthermore, brain iron accumulation has been reported in hypotransferrinemic mice [132] and β-thalassemia patients [133,134]. In fact, the transport into the brain of ^59^Fe-labelled NTBI (in the form of FeCl_3_ or ferric ammonium citrate) has been shown in a mouse model [135]. However, it should be noted that, given the high Tf saturation, NTBI is a physiological form of iron in the cerebrospinal fluid [136] and it is actively taken up by microglia, astrocytes and neurons [137].

## 8. Final Remarks

More than four decades have passed since the discovery of NTBI, but its biochemical characterization remains elusive. The term, NTBI, refers to a heterogenous pool of iron species, resulting from the binding Fe^3+^ to ligands in the blood serum. Currently, the best working chemical model assumes that the main serum ligands are citrate anions and HSA. The most recent results suggest that these compartments form a fine-tuned equilibrium dependent on Fe^3+^ concentration, iron:citrate molar and HSA modifications. At low NTBI levels, Fe^3+^ is expected to be mostly associated with HSA, with mononuclear [Fe(Cit)_2_]^5−^ being the predominant LMW species. At the higher bound of pathophysiological NTBI concentrations (>10 uM), most of the NTBI will be associated with citrate, with mononuclear [Fe(Cit)_2_]^5−^ being the predominant species and polynuclear complexes ([Fe_2_(Cit)_2_]^2−^ or [Fe_3_(Cit)_3_]^3−^) representing 20% to 40% of the non-protein-bound iron. Recently, Dziuba et al. [138] reported that LMW iron in healthy plasma samples was not bound to citrate. Under these conditions, however, Fe^3+^-citrate is not expected to exist, as the predominant apo-Tf is predicted to out-compete citrate for the binding of such iron. These iron species, occur in the nM range and, most probably, are transient species rapidly cleared by Tf.

NTBI speciation will define the toxicity pathways of these iron species, by modulating their redox reactivity [84], or preferential cellular uptake routes [39]. In particular, the biological implications of iron binding by HSA have been less explored, but they are certain to play a decisive role in the biochemistry of NTBI. HSA may modulate the pro-oxidant character of NTBI in the plasma [84,139], and it facilitates iron donation to apo-Tf [140]. HSA also offers an alternative route for cellular uptake, through endocytosis [141]. Furthermore, HSA post-translational modifications offer a possible explanation for disease-specific modulation of NTBI reactivity and toxicity [66]. This is particularly relevant in a context where similar modifications may also impact the ability of Tf to bind iron [6]. Finally, it is relevant to note that HSA-bound iron is underestimated by the NTA-based assay [66].

Despite the progress achieved in the past years, a better understanding of NTBI speciation is a crucial step towards defining clinically relevant isoforms and the development of reliable analytical assay.

## Data Availability

Not applicable.
